# Social Media Surveillance in Schools: Rethinking Public Health Interventions in the Digital Age

**DOI:** 10.2196/22612

**Published:** 2020-11-12

**Authors:** Colin Burke, Cinnamon Bloss

**Affiliations:** 1 University of California, San Diego La Jolla, CA United States

**Keywords:** social media, surveillance, privacy, public health, students, schools, social media surveillance, school safety, mental health, adolescents

## Abstract

Growing public concern about student safety and well-being has led schools and school districts to contract private companies to implement new technologies that target and surveil students’ activity on social media websites. Although innovative solutions for addressing student safety and health are needed, it is unclear whether the implementation of social media surveillance in schools is an effective strategy. Currently, there is no evidence to support the claims made by social media surveillance companies, as well as the schools that hire them, that these technologies can address the myriad of public health issues facing today’s students. Instead, these digital surveillance systems may only serve to exacerbate the problems that youth—especially those from historically marginalized groups—already face.

Over the last two decades, schools have faced several concerning trends related to student safety and well-being, including acts of violence, cyberbullying, and adolescent suicidality. The number of shooting incidents in K-12 schools, for instance, has increased since 1970, with a record 97 shooting incidents in 2018 alone [1]. Furthermore, emerging data indicate worrisome trends related to online bullying [2] and adverse mental health outcomes, including increased rates of adolescent suicide [3]. Since social media websites have become a primary medium for students to express their thoughts, views, and feelings, social media has also been increasingly seen as a potential site for intervention and prevention of these public health threats.

Concern about the safety and security of schools and students has led to an increasing number of US schools and school districts hiring private companies to monitor students’ social media activity [4]. Companies that provide social media surveillance services purportedly have the ability to identify and report any public social media posts made by students that fall under predetermined categories of concern. The first and most widely covered case of social media surveillance took place in the Glendale School District in California, where the suicide of a student in 2013 prompted the district to contract an external company to monitor and analyze students’ social media accounts. Since then, thousands of schools and school districts have hired companies to provide social media surveillance services. Although innovative solutions for addressing students’ safety and health are needed, it is unclear whether the implementation of social media surveillance in schools is an effective strategy. Despite the increased implementation of social media surveillance in schools, the public is generally unaware that these services are so widely utilized. This lack of awareness means that there has also been little to no consideration of the consequences of implementing these social media surveillance technologies. More thoughtful debate and study are needed to bring about greater public and scholarly attention to the use of these technologies and to better understand the potential implications of their use in schools.

Schools and school districts are not alone in their attempts to monitor students’ social media activity. Most recently, the state of Florida contracted a private technology firm called FivePoint Solutions to monitor and analyze the Florida Schools Safety Portal (FSSP), which consolidates data from Florida’s Department of Education, Department of Children and Families, Department of Law Enforcement, Department of Juvenile Justice, and local law enforcement, as well as students’ posts from social media websites. The FSSP, which is currently in use by all Florida public schools, aims to allow “school threat assessment teams to identify, assess and provide intervention services for individuals whose behavior may pose a threat to themselves or others” and “report suspicious activities to the proper authorities within the school district” [5].

Taken at face value, social media surveillance services may present an opportunity for schools to increase their awareness of students’ online activity, as well as better identify and prevent potential instances of harm that may otherwise go unreported. The integration of multiple databases (eg, the FSSP [Florida Schools Safety Portal]) provides school officials with a large amount of information about their students. Theoretically, this helps school officials to make more informed decisions about potential threats to the safety of their students. A recent study by the University of Chicago Crime Lab found that social media surveillance carried out by Chicago public school officials, in conjunction with targeted interventions, led to positive outcomes for Chicago public school students, including lower risk of exposure to out-of-school shooting incidents, fewer misconduct incidents, fewer out-of-school suspensions, and higher rates of school attendance [6]. Although this report suggests that social media surveillance may have some utility, there is currently no evidence that social media surveillance is able to effectively address the public health issues that many social media surveillance services, as well as the schools that use them, claim to be targeting, such as cyberbullying, students’ mental health, and violence in schools.

The deployment of social media surveillance technologies in school settings also raises some concerns. First, while students’ social media posts are public, educational professionals are certainly not in students’ imagined audience. Considerable harm can be done through the act of sharing students’ online activity with school administrators who hold positions of power over students. School administrators are not immune to bias, and simply seeing this information can potentially influence their perceptions of students in a negative way, whether consciously or subconsciously. Students may also experience embarrassment or shame as a result of knowing that their online activity was accessed by and distributed to an unintended audience, potentially exacerbating any existing mental health issues (eg, depression and anxiety). In addition, the language and culture of today’s students (ie, memes) may not be easily understood and interpreted by older school officials, who are faced with the difficult task of translating and interpretating students’ online activity to identify potential threats and harm. Thus, there is a considerable risk of false positives due to this cross-generational cultural barrier, which may lead to the unwarranted punishment of students, as well as general distrust in school administrators and the institutions they represent.

Second, social media surveillance technologies are vulnerable to algorithmic biases that may disproportionately target particular individuals or groups. The FSSP, for example, collects information from several Florida state-wide databases, and biases within these databases must be taken into account. The dynamics of inclusion and exclusion within these databases can have a significant impact on who is labeled a threat and subjected to increased monitoring and surveillance. This becomes especially problematic when some databases are inherently overrepresented by marginalized racial and socioeconomic groups. Black youth, for example, represent over half of the juvenile arrests in the Florida Department of Juvenile Justice database [7], which is where crime and other adolescent misconduct are logged. The overrepresentation of Black youth in this database could lead an algorithm to produce biased findings, such as considering Black youth a greater threat than members of other racial groups. Similarly, data from the Florida Department of Children and Families are more likely to include students from lower socioeconomic backgrounds. The use of these incomplete databases means that threat assessment and the resulting target of surveillance systems may be primarily directed toward students in marginalized groups who are overrepresented in such databases. In contrast, students from more privileged racial and socioeconomic groups, who these databases tend to exclude, may be less likely to be subjected to further surveillance. Algorithms are only as good as the data they are trained on; feeding algorithm-biased data leads to biased outcomes [8-10], which, in this case, could mean disproportionately targeting Black youth and students from lower socioeconomic backgrounds.

The Florida case highlights the possible dangers that are inherent in relying upon digital technological systems and so-called “big data” to inform public health interventions without careful consideration of the limitations and biases that exist within these systems. Although innovative approaches to student safety and well-being are needed, it is unclear whether the implementation of social media surveillance in schools is an effective strategy. Despite their potential utility, there is currently no compelling empirical evidence to support the claims made by social media surveillance companies, as well as the schools that hire them, that social media surveillance technologies can effectively address the public health threats facing today’s students, such as cyberbullying, adolescent suicidality, and acts of violence. Instead, these digital surveillance systems may only serve to exacerbate the problems that youth—particularly those from historically marginalized groups—already face. Furthermore, these public health interventions are being carried out on a massive scale with little public awareness and regard for the consequences of their implementation. The potential pitfalls of social media surveillance mean that we, as a society, must engage in a more extensive dialogue to focus our attention on the use and effects of these technologies.

## Abbreviations

FSSP: Florida Schools Safety Portal
